# Introducing *JPGN Reports*

**DOI:** 10.1097/PG9.0000000000000001

**Published:** 2020-07-17

**Authors:** Melvin B. Heyman, Dominique C. Belli

**Affiliations:** Professor of Pediatrics, UCSF Benioff Children’s Hospital, University of California, San Francisco, San Francisco, CA; Professor of Pediatrics, Welcome Center HUG-Unige-HeSSo, University of Geneva, Geneva, Switzerland

**Keywords:** pediatrics, gastroenterology, hepatology, nutrition, pancreas, liver

We are pleased to announce a new online, peer-reviewed, open access journal, *JPGN Reports*, a joint publication by the North American Society of Pediatric Gastroenterology, Hepatology, and Nutrition (NASPGHAN) and the European Society of Paediatric Gastroenterology, Hepatology, and Nutrition (ESPGHAN). *JPGN Reports* will be a popular venue for publication, especially for case reports and clinical studies related to pediatric gastroenterology, hepatology, and nutrition.

*JPGN Reports* aims to publish novel case reports, images and videos, original articles, clinical trials protocols, review articles, short communications, letters to the editor, selected meeting proceedings, editorials, and commentaries on all aspects of clinical and translational research, educational, and public health issues in the area of pediatric gastroenterology, hepatology, and nutrition. *JPGN Reports* also welcomes novel exploratory hypothesis-generating research, qualitative and quantitative epidemiologic research, studies of novel mechanisms and methodologies including public health interventions, hypothesis-generating small studies, methods papers, and translational research applicable for pediatric pathogenesis and physiology in health and disease.

We welcome your submissions to our new journal. Manuscripts may be submitted to *JPGN Reports* directly to the website: https://www.editorialmanager.com/jpgnreports/default.aspx.

